# The relationship between blood lipid and risk of psoriasis: univariable and multivariable Mendelian randomization analysis

**DOI:** 10.3389/fimmu.2023.1174998

**Published:** 2023-06-22

**Authors:** Zeng-Yun-Ou Zhang, Zhong-Yu Jian, Yin Tang, Wei Li

**Affiliations:** ^1^ Department of Dermatology, West China Hospital, Sichuan University, Chengdu, China; ^2^ Department of Urology, Institute of Urology (Laboratory of Reconstructive Urology), West China Hospital, Sichuan University, Chengdu, China; ^3^ Department of Dermatology & Rare Disease Center, West China Hospital, Sichuan University, Chengdu, Sichuan, China

**Keywords:** blood lipid, psoriasis, Mendelian randomization, risk factor, GWAS - genome-wide association study

## Abstract

**Background:**

Psoriasis is a chronic inflammatory skin disease. Dyslipidemia may be a risk factor of psoriasis. But the causal relationship between psoriasis and blood lipid still remains uncertain.

**Methods:**

The two data of blood lipid were obtained from UK Biobank (UKBB) and Global Lipid Genetics Consortium Results (GLGC). The primary and secondary database were from large publicly available genome-wide association study (GWAS) with more than 400,000 and 170,000 subjects of European ancestry, respectively. The psoriasis from Finnish biobanks of FinnGen research project for psoriasis, consisting of 6,995 cases and 299,128 controls. The single-variable Mendelian randomization (SVMR) and multivariable Mendelian randomization (MVMR) were used to assess the total and direct effects of blood lipid on psoriasis risk.

**Results:**

SVMR estimates in primary data of blood lipid showed low-density lipoprotein cholesterol (LDL-C) (odds ratio (OR): 1.11, 95%, confidence interval (CI): 0.99−1.25, *p* = 0.082 in stage 1; OR: 1.15, 95% CI: 1.05−1.26, *p* = 0.002 in stage 2; OR: 1.15, 95% CI: 1.04−1.26, *p* = 0.006 in stage 3) and triglycerides (TG) (OR: 1.22, 95% CI: 1.10−1.35, *p* = 1.17E-04 in stage 1; OR: 1.15, 95% CI: 1.06−1.24, *p* = 0.001 in stage 2; OR: 1.14, 95% CI: 1.05−1.24, *p* = 0.002 in stage 3) had a highly robust causal relationship on the risk of psoriasis. However, there were no robust causal associations between HDL-C and psoriasis. The SVMR results in secondary data of blood lipid were consistent with the primary data. Reverse MR analysis showed a causal association between psoriasis and LDL-C (beta: -0.009, 95% CI: -0.016− -0.002, *p* = 0.009) and HDL-C (beta: -0.011, 95% CI: -0.021− -0.002, *p* = 0.016). The reverse causation analyses results between psoriasis and TG did not reach significance. In MVMR of primary data of blood lipid, the LDL-C (OR: 1.05, 95% CI: 0.99–1.25, *p* = 0.396 in stage 1; OR: 1.07, 95% CI: 1.01–1.14, *p* = 0.017 in stage 2; OR: 1.08, 95% CI: 1.02–1.15, *p* = 0.012 in stage 3) and TG (OR: 1.11, 95% CI: 1.01–1.22, *p* = 0.036 in stage 1; OR: 1.09, 95% CI: 1.03–1.15, *p* = 0.002 in stage 2; OR: 1.07, 95% CI: 1.01–1.13 *p* = 0.015 in stage 3) positively correlated with psoriasis, and there had no correlation between HDL-C and psoriasis. The results of the secondary analysis were consistent with the results of primary analysis.

**Conclusions:**

Mendelian randomization (MR) findings provide genetic evidence for causal link between psoriasis and blood lipid. It may be meaningful to monitor and control blood lipid level for a management of psoriasis patients in clinic.

## Introduction

Psoriasis is a chronic inflammatory skin disease. It is typically reported to affect approximately 1%–3% of the people every year (1). According to most researches, the white individuals have higher morbidity rates than other ethnic groups (2, 3). However, psoriasis is not just a skin disease, it is actually related to the occurrence of other disorders, including metabolic syndrome (MetS), rheumatological, cardiovascular and inflammatory bowel disease (4). This disease causes great physical and psychological burden for patients (2). Risk factors for psoriasis are diverse, such as cardiovascular disease, MetS, diabetes mellitus, obesity, dyslipidemia and hypertension and so on (5, 6). These risk factors are a challenge to the treatment and management of psoriasis (7). Thus, it is meaningful to find modifiable risk factors to prevent psoriasis.

MetS is one of the most common comorbidities in psoriasis, and characterized by obesity, hypertension, diabetes mellitus and hyperlipidemia (8, 9). Among these, dyslipidemia is one of typical manifestations. An increasing number of studies suggested that dyslipidemia can be observed in most patients with psoriasis (10). The most common manifestations are elevated the serum cholesterol, low-density lipoprotein cholesterol (LDL-C) and triglycerides (TG) levels, and lowered value of high-density lipoprotein cholesterol (HDL- C) (11–13). Many comprehensive studies revealed an increasing graded relationship between MetS and severity of psoriasis (4, 14, 15). Dyslipidemia can also increase the risk of developing psoriasis comorbidities, such as MetS, cardiovascular disease and non-alcoholic fatty liver disease (NAFLD). In turn, the comorbidities (such as MetS) may aggravate dyslipidemia and form a vicious circle (16–19). Lotus Mallbris et al. found dyslipidemia in psoriasis may be due to genetic influence rather than acquired (12). However, another study demonstrated psoriasis is an independent risk factor for dyslipidemia (20). There still remain controversial whether blood lipid associated with increased risk of psoriasis or the psoriasis affects blood lipid level.

For the limitations of observational studies, many studies cannot determine the temporal relationship between psoriasis onset and dyslipidemia. While psoriasis may in turn affect changes in blood lipid, which makes the causal relationship between psoriasis and blood lipid more unclear. Considering the controversies, Mendelian randomization (MR) can be applied for causal inference between psoriasis and dyslipidemia. The MR is widely used to explore the causality between risk factors and diseases (21, 22), employs single-nucleotide polymorphisms (SNPs) as genetic tools and reliably estimates their effects on the outcomes of interest. It is a genetic method that assesses causality under certain assumptions, independent of confounders in the environment. Thus, we used MR to evaluate the causal relationships between psoriasis and blood lipid, hoping to provide genetic evidence for resolving the existing controversy.

## Methods

### Study design

The MR design consisted of the analysis of two blood lipid genome-wide association study (GWAS) databases, namely the primary database analysis and the secondary database analysis. The selection of genetic instrumental variables for blood lipid is mainly divided into three stages, each stage will select suitable SNPs for corresponding MR analysis. To improve the credibility of the results, we performed MR analysis for SNPs of blood lipid in each stage, including single-variable Mendelian randomization (SVMR) and multivariable Mendelian randomization (MVMR). Finally, we performed a reversal MR analysis to explore the effect of psoriasis on blood lipid. This reversal causation analysis will help determine whether psoriatic disease status could affect the levels of blood lipid.

### Data resources

We obtained the GWAS for blood lipid from two independent database, namely UK Biobank (UKBB) and Global Lipid Genetics Consortium Results (GLGC). The primary database was form UKBB. There were 441,016 for TG, 403,943 for HDL-C, and 440,546 for LDL-C included in GWAS. Detailed GWAS methods were provided in original article (23). The secondary database was from GLGC. The TG (N = 177,861), HDL-C (N = 187,167) and LDL-C (N = 173,082) were also from 188,577 European-ancestry individuals (24). Outcome data for psoriasis were obtained from Finnish biobanks of FinnGen research project, consisting of 6,995 cases and 299,128 controls. The details for used data sources were presented in **Supplementary Table 1**. For the reversal causation analysis, the genetic instruments consisting of 36 SNPs for psoriasis was derived from a GWAS meta-analysis, involving 10,588 cases and 22,806 controls in total (25).

### Outcome definition

Psoriasis definition in FinnGen R7 were demonstrated online (https://r7.risteys.finngen.fi/phenocode/L12_PSORIASIS). Briefly, it was diagnosed using the Hospital Discharge: ICD-10 L40 and Cause of death: ICD-10 L40. After a series of filtering, 6995 of cases (3677 Female and 3318 Male) were retained in GWAS.

### Instrument selection

**Figure 1:**
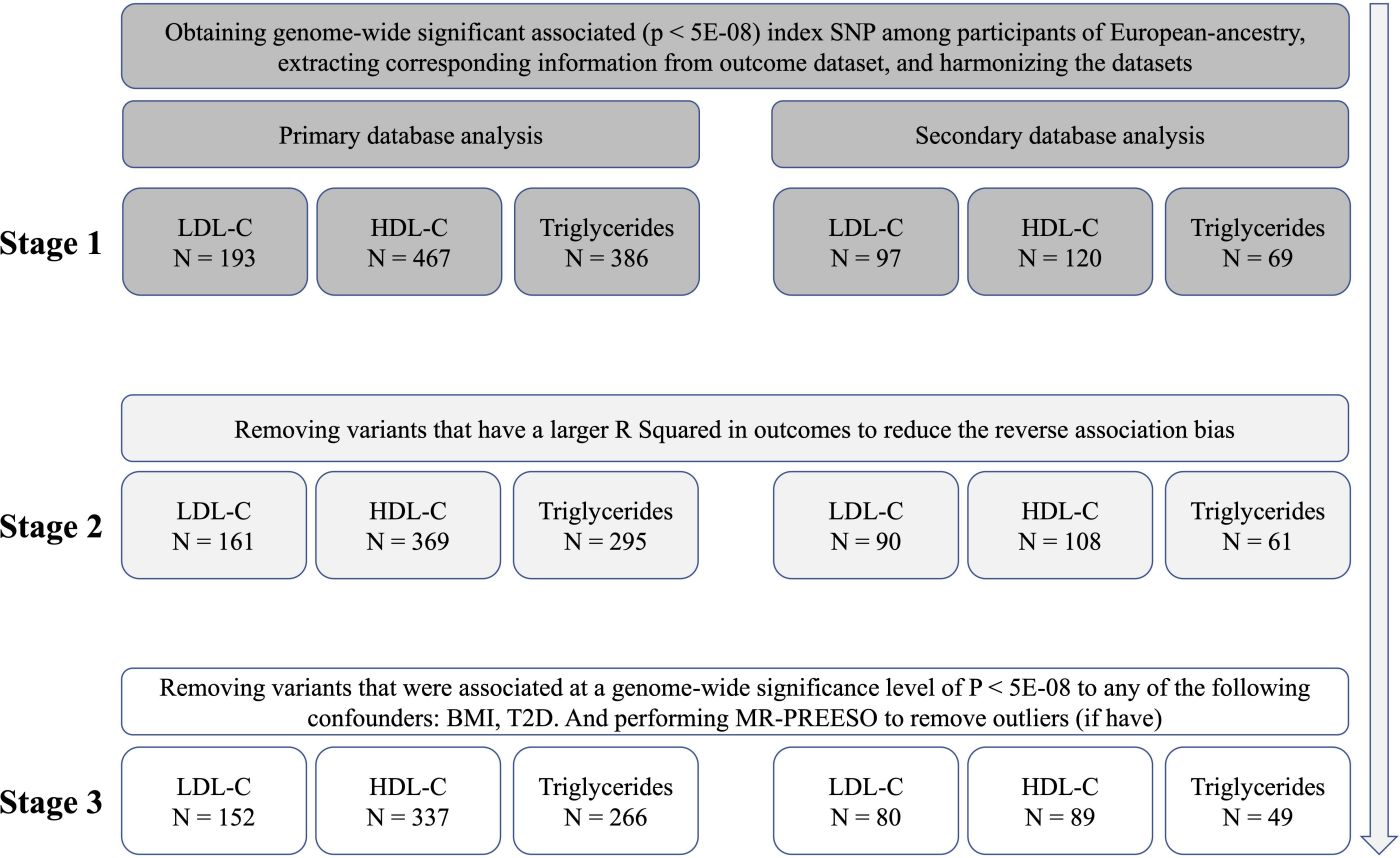
The identification of genetic instruments, and data and MR methods used for analyses. N, numbers of SNPs; LDL-C, Low-density lipoprotein cholesterol; HDL-C, high-density lipoprotein cholesterol; SNP, single-nucleotide polymorphisms; MR, Mendelian randomization; BMI, Body Mass Index; T2D, Type 2 Diabetes.

For primary database analysis, we firstly selected independent SNPs from a recent reported GWAS which including 220 (LDL-C), 534 (HDL-C), and 440 (TG) independent SNPs (23). After extracting corresponding information from outcome dataset and harmonizing the exposure and outcome data, 193 SNPs for LDL-C, 193 SNPs for HDL-C and 193 SNPs for TG were retained. We would perform MR after this step, called stage 1 analysis. Secondly, to reduce the reverse association bias, we remove variants that have a larger R Squared in outcomes and perform the stage 2 analysis. Thirdly, we examined the instrumental variables’ (IVs) association with potential confounders, and removed variants that were associated at a genome-wide significance level of P < 5E-08 to any of the following confounders: type 2 diabetes (T2D), BMI (**Supplementary Table 2**), and performed MR-PREESO to remove outliers (if have). After this step, we performed MR again using the retained SNPs in stage 3 analysis. To make our results more robust, we performed secondary database analysis as a replication using another GWAS enrolling different populations for lipid traits (24). Initial independent SNPs were selected from online (p < 5E-08, linkage disequilibrium < 0.01) (Freq.A1.1000G.EUR). The fowling steps were the same as the primary database analysis. The detailed SNP filtering and corresponding number of SNPs was presented in **Figure 1**. The details of SNPs were presented in **Supplementary Data 1**. To avoid the effect of weak instrumental variable bias, we used the F statistic to assess the strengths of IVs. After calculating, the F statistics of all SNPs in every stage of both databases were greater than 10, indicating a smaller possibility of weak instrumental variable bias (**Supplementary Table 2**).

### MR assumption

The two-sample MR design is based on three main hypotheses of classical assumptions (26, 27):(1) the IVs with blood lipid is closely associated with the exposure of interest; (2) There are no confounders between IVs and outcomes; (3) IVs directly affect outcomes through the exposure of interest and not through other pathways. We tried to exclude some confounding factors related to blood lipid. Moreover, by rigorous screening for IVs, we reduced the weak association between potential confounders and genetic variations. Effected by horizontal pleiotropy, genetic variation may affect outcomes in ways other than exposure of interest. In this MR study, we used three MR methods to estimate the robust effects, including inverse variance weighted (IVW), Mendelian randomization-Egger (MR–Egger), weighted median, and weighted methods. We performed MR-egger intercept analysis on SNPs at each stage in primary and secondary database analysis, the results indicated there was no pleiotropy (Egger intercept *p* value > 0.05). The details are presented in **Supplementary Tables 3**, **4**.

### Statistical analysis

In MR analysis, the IVW and MR–Egger methods were used as the main analysis method. MR–Egger, IVW, weighted median, and weighted methods are used to estimate the robust effects if genetic variants have pleiotropic effects. The weighted median method provides reliable evidence when at least half of the valid instrumental variables have no pleiotropic effects. The MR–Egger regression provides consistent estimates when 100% of genetic variants are invalid IVs.

The packages Two-Sample MR and MVMR in R v.4.0.3 (www.r-project.org) are used to analysis. The online tool is used to perform power calculations (http://cnsgenomics.com/shiny/mRnd/).

## Results

### Univariable MR analysis of blood lipid on psoriasis risks

#### LDL-C as exposure

We analyzed SNPs for each stage of LDL-C from primary and secondary databases. Overall, we found a highly robust causal associations between LDL-C and psoriasis outcomes. In primary database analysis, we observed evidence for genetically predicted LDL-C through IVW method (OR: 1.11, 95% CI: 0.99–1.25, *p* = 0.082 in stage 1; OR: 1.15, 95% CI: 1.05–1.26, *p* = 0.002 in stage 2; OR: 1.15, 95% CI: 1.04–1.26, *p* = 0.006 in stage 3). In secondary database analysis, LDL-C still had a strongly causal association with psoriasis (OR: 1.09, 95% CI: 0.99–1.94, *p* = 0.081 in stage 1; OR: 1.10, 95% CI: 1.02–1.18, *p* = 0.009 in stage 2; OR: 1.12, 95% CI: 1.03–1.21, *p* = 0.008 in stage 3) through IVW method (**Table 1**). Furthermore, we used three other MR methods to analyze the SNPs, and the results were basically consistent with the IVW analysis (**Supplementary Tables 3**, **4**). Sensitivity analysis showed a consistent trend across the four MR methods (**Figure 3**). MR analysis of both databases revealed a positive association between LDL-C and psoriasis. This indicated that LDL-C was a risk factor for psoriasis.

**Table 1:** 2-sample MR results for associations between lipid traits and psoriasis using IVW method.

Exposure	Analysis stage	N-SNPs	OR	95%LCI	95%UCI	pval	Qhet (pval)	Egger_intercept (pval)
Primary database analysis								
LDL-C	Stage 1	193	1.110	0.987	1.248	0.082	6.48E-12	0.206
	Stage 2	161	1.152	1.054	1.259	0.002	0.999	0.083
	Stage 3	152	1.145	1.039	1.261	0.006	1.000	0.054
HDL-C	Stage 1	467	0.917	0.825	1.020	0.112	2.65E-41	0.265
	Stage 2	369	0.954	0.885	1.029	0.226	1.000	0.886
	Stage 3	337	0.969	0.896	1.048	0.426	1.000	0.869
TG	Stage 1	386	1.222	1.103	1.353	1.17E-04	3.92E-18	0.272
	Stage 2	295	1.146	1.057	1.243	0.001	1.000	0.372
	Stage 3	266	1.140	1.047	1.241	0.002	1.000	0.210
Secondary database analysis								
LDL-C	Stage 1	97	1.087	0.990	1.194	0.081	2.64E-06	0.826
	Stage 2	90	1.099	1.024	1.180	0.009	0.580	0.619
	Stage 3	80	1.117	1.029	1.212	0.008	0.433	0.426
HDL-C	Stage 1	120	0.926	0.837	1.025	0.139	4.02E-05	0.830
	Stage 2	108	0.926	0.854	1.005	0.065	0.809	0.538
	Stage 3	89	0.933	0.856	1.016	0.111	0.651	0.581
TG	Stage 1	69	1.224	1.063	1.409	0.005	4.02E-07	0.555
	Stage 2	61	1.173	1.062	1.296	0.002	0.731	0.685
	Stage 3	49	1.137	1.018	1.271	0.023	0.544	0.496

N-SNPs, numbers of single-nucleotide polymorphisms; OR, odds ratio; CI, confidence interval; LDL-C, Low-density lipoprotein cholesterol; HDL-C, high-density lipoprotein cholesterol; TG, triglycerides.

#### HDL-C as exposure

**Figure 2:**
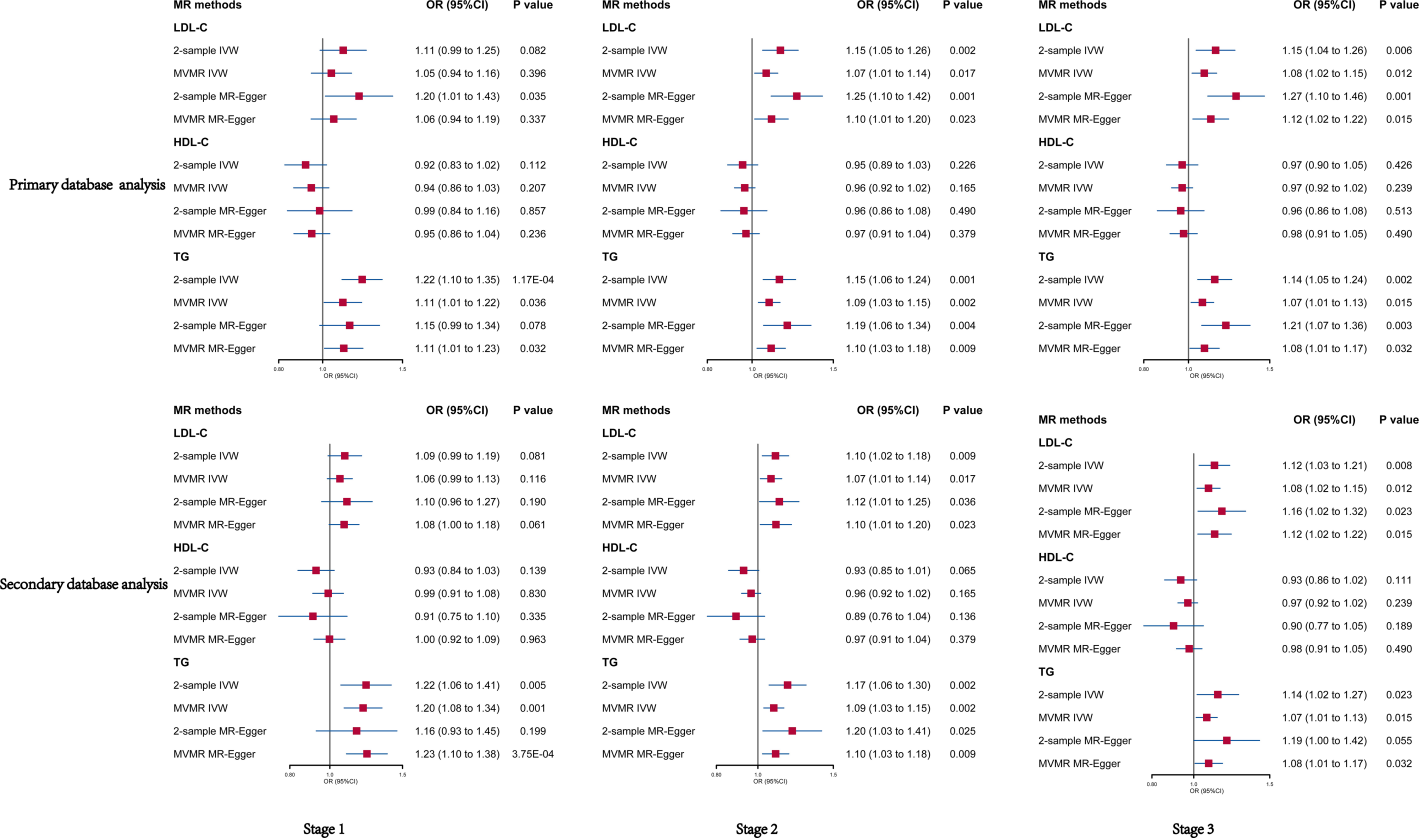
Univariable and multivariable MR of the effect of LDL-C, HDL-C and TG on psoriasis in primary and secondary data, respectively. OR, odds ratio; CI, confidence interval; LDL-C, Low-density lipoprotein cholesterol; HDL-C, high-density lipoprotein cholesterol; TG, triglycerides.

**Figure 3:**
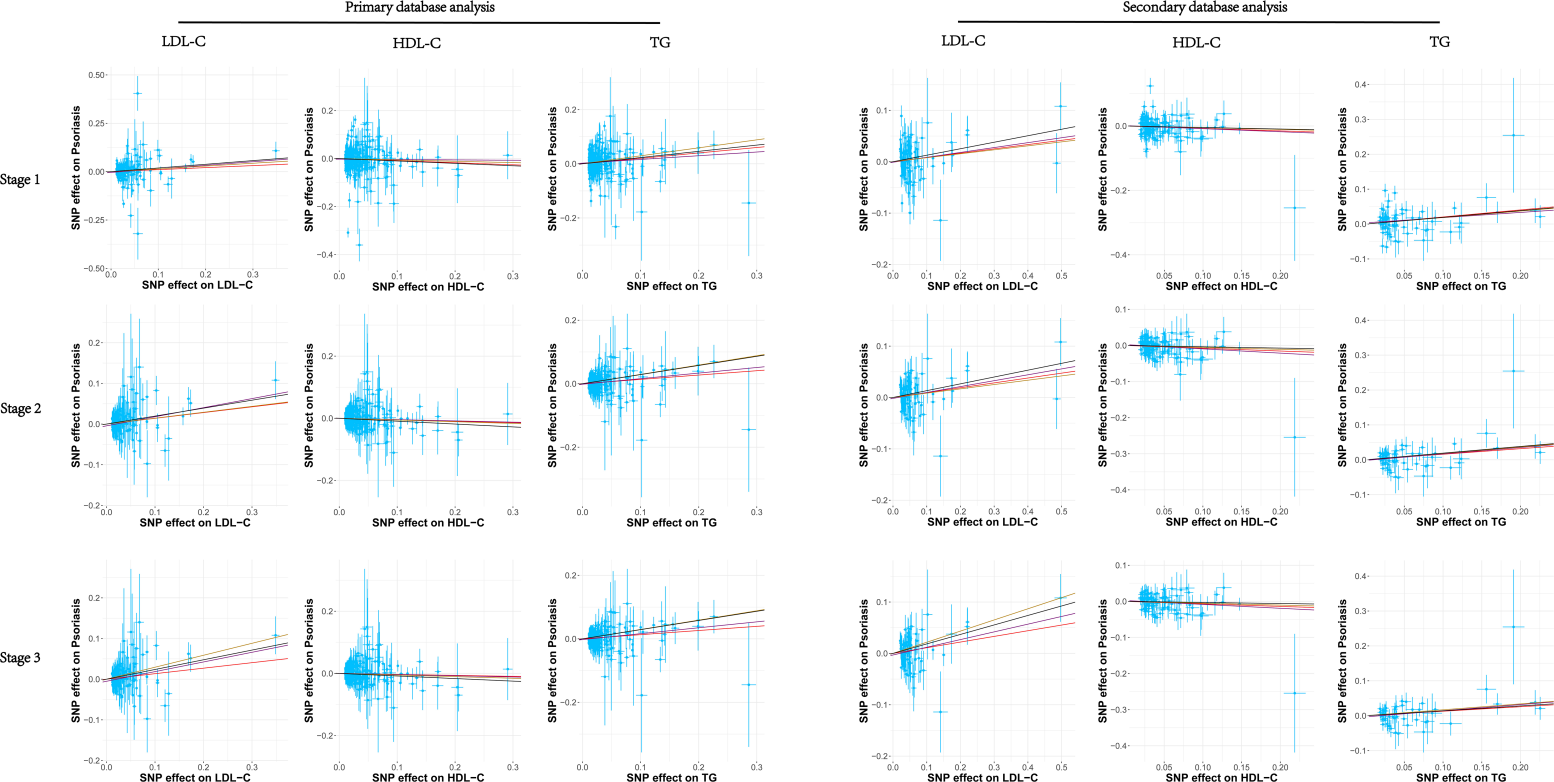
The scatter plots of all MR test results in primary database and secondary database analysis, respectively represents the IVW, MR-Egger, Weighted median, and Weighted mode effect, respectively. The slope of the line represents the MR effect size. The red, purple, yellow, and black line. LDL-C, Low-density lipoprotein cholesterol; HDL-C, high-density lipoprotein cholesterol; TG, triglycerides; SNP, single-nucleotide polymorphisms.

Overall, we did not observe causal association between HDL-C and psoriasis in primary database and secondary database analysis. No significant result was found through IVW and MR egger method analysis in each stage from primary and secondary database (**Figure 2**). To exclude the effect of horizontal pleiotropy, using Weighted median and Weighted mode methods to analyze all SNPs from the two databases, there was no horizontal pleiotropy and significant result (**Supplementary Tables 3**, **4**). Finally, the sensitivity analysis of the four MR analysis methods showed a robust consistent trend (**Figure 3**). Therefore, we considered there was no highly robust causal associations between HDL-C and psoriasis.

#### TG as exposure

We observed a strong causal association between TG and psoriasis in primary database and secondary database analysis (**Figure 2**). In primary database analysis, IVW method showed TG (OR: 1.22, 95% CI: 1.10–1.35, *p* = 1.17E-04 in stage 1; OR: 1.15, 95% CI: 1.06–1.24, *p* = 0.001 in stage 2; OR: 1.14, 95% CI: 1.05–1.24, *p* = 0.002 in stage 3) had a significant association with psoriasis in each stage. In secondary database analysis, TG had a robust association with psoriasis (OR: 1.22, 95% CI: 1.06–1.41, *p* = 0.005 in stage 1; OR: 1.17, 95% CI: 1.06–1.30, *p* = 0.002 in stage 2; OR: 1.14, 95% CI: 1.02–1.27, *p* = 0.02 in stage 3) through IVW method (**Table 1**). The results of MR egger method, Weighted median and Weighted were still strongly association between TG and psoriasis (**Supplementary Tables 3**, **4**). The sensitivity analysis showed a consistent trend (**Figure 3**). These results revealed the TG was a strong risk factor for psoriasis. Elevated TG levels may increase risk of psoriasis in European population.

### Multivariable MR analysis of blood lipid on psoriasis risks

In general, the results of MVMR analysis in primary and secondary database indicated that LDL-C and TG were risk factors for psoriasis, but HDL-C had no association with psoriasis. In primary database analysis, the results of IVW method showed that LDL-C (OR: 1.05, 95% CI: 0.99–1.25, *p* = 0.396 in stage 1; OR: 1.07, 95% CI: 1.01–1.14, *p* = 0.017 in stage 2; OR: 1.08, 95% CI: 1.02–1.15, *p* = 0.012 in stage 3) and TG (OR: 1.11, 95% CI: 1.01–1.22, *p* = 0.036 in stage 1; OR: 1.09, 95% CI: 1.03–1.15, *p* = 0.002 in stage 2; OR: 1.07, 95% CI: 1.01–1.13 *p* = 0.015 in stage 3) positively correlated with psoriasis, and there had no correlation between HDL-C and psoriasis (OR: 0.94, 95% CI: 0.86–1.03, *p* = 0.207 in stage 1; OR: 0.96 95% CI: 0.92–1.02, *p* = 0.165 in stage 2; OR: 0.97 95% CI: 0.92–1.02, *p* = 0.239 in stage 3) (**Figure 2**). The results of the secondary database analysis were consistent with the results of primary database analysis. The details were presented in **Figure 1**. In MR egger method of primary database and secondary database analysis, the association between LDL-C and TG with risk of psoriasis were still significant in each stage. It also showed that HDL-C was not significantly associated with psoriasis (**Figure 2**).

### Psoriasis as exposure and blood lipid as outcome

We performed a reversal MR analysis to explore the effect of psoriasis on blood lipid. We found a causal association between psoriasis and LDL-C (beta: -0.009, 95% CI: -0.016– -0.002, *p* = 0.009) and HDL-C (beta: -0.011, 95% CI: -0.021– -0.002, *p* = 0.016). The reverse causation analyses results between psoriasis and TG did not reach significance. The details are presented in **Supplementary Table 5**.

## Discussion

Using 2-sample and MVMR analysis, we found the strong genetic evidence that increasing the serum level of LDL-C and TG was associated with higher risk of psoriasis. The reverse MR revealed the status of psoriasis could affect the level of LDL-C and HDL-C. Overall, we found the genetic association between dyslipidemia and the risk of incident psoriasis were significant through MR method analysis.

In most clinical observational studies, dyslipidemia can be found in patients with psoriasis (28–30). As for previous clinical observational studies on LDL-C and psoriasis, elevated LDL-C levels were common. A big meta-analysis concluded that VLDL and LDL have been proved to be significantly higher in psoriatic patients (31). Other observational studies also reached the same conclusion (16, 32). In addition, in some observational studies of psoriatic arthritis (PSA), the serum TG, HDL-C, TC, and LDL-C have no significant association with psoriatic arthritis (33–35). The relationship between psoriasis and blood lipid is still unclear due to the evidence of publication bias and confounding factors in the environment. Our MR analysis results showed a strong genetic evidence show that the increase of LDL-C was associated with increased risk of psoriasis. Mehta et al. (36) have reported both the structure and function of lipoprotein were altered in psoriasis, with increased LDL-C particle concentration and deceased size. Orem A et al. (37) find a positive relationship between the serums level of autoantibodies against ox-LDL and severity of psoriasis. Of note, oxidized low-density lipoprotein (ox-LDL) is considered a marker to assess the severity of psoriasis. A study found an elevation in the ratio of anti-ox-LDL to ox-LDL could serve as a composite parameter reflecting the total oxidative lipoprotein burden in patients with psoriasis (38). Another prospective longitudinal cohort study has investigated a dependent association between elevated lectinlike ox-LDL receptor-1 and psoriasis severity (39). So far, the lipoprotein metabolism and dyslipidemia may be associated with development of psoriasis independent of hyperlipidemia status, but the actual underlying mechanisms are still unclear. We observed strong evidence for a causal link between LDL and psoriasis, and the reverse causality showed negative correlation. This may be associated with higher levels of LDL-C producing more ox-LDL. However, the LDL-C effects of mechanism by psoriasis required in-depth study.

For HDL-C, the relationship between it and psoriasis became more complex. An observational study shows the psoriasis patients have significantly higher very-low-density lipoprotein (VLDL) and HDL-C (12). However, in a population-based, cross-sectional study revealed the cholesterol, LDL-C, TG, and alanine aminotransferase are significantly higher in psoriasis. But the serum HDL-C has no association with psoriasis (13). In other large clinical observational studies, no clear relationship has been shown between the type of dyslipidemia and psoriasis. In a large cross-sectional study, there is no substantial association for total cholesterol (TC), TG and HDL-C (40). Another cross-sectional study showed the same results (16). We observed there was no causal relationship between HDL-C and psoriasis risks. Some studies have demonstrated that lipid function depend more on their structural and functional alterations than level of lipid in psoriasis (36, 41). Holzer M et al. (42) also reveal the lipid composition of HDL-C is altered from 15 patients with psoriasis. Even the efflux capacity of HDL-C and cholesterol were decreased. Furthermore, the similar changes of lipoprotein also occur in children with psoriasis (41). These findings indicate the transport defects of reverse cholesterol in HDL-C start early in life. Our MR results showed no significant difference between HDL-C levels and psoriasis. However, a positive reverse causality association was found between the psoriasis and HDL-C. Hereditarily, this suggested the level of HDL-C maybe have no correlation to the onset of psoriasis. But in turn, psoriasis may affect the structural and functional alterations of HDL-C.

Elevated TG is one of the diagnostic criteria of MetS, and it is also a risk factor for psoriasis comorbidities, such as cardiovascular diseases (43). A across-sectional controlled study demonstrates the serum cholesterol, TG and LDL-C were significantly higher between psoriasis patients with control group (20). Some observational studies reveal a similar result that TG is elevated in patients with psoriasis (13, 44). However, other studies find that TG have no significant difference between PsA group and controls group (45, 46). In addition to the LDL oxidation, S. Kaur et al. (47) suggested the TG level may be the mechanisms behind the psoriasis. But the relationship of pathogenesis between TG and psoriasis was still unclear due to lack of relevant research. In this MR study showed a strong positive correlation between TG and psoriasis, and provided genetic evidence for a link between TG and psoriasis. This risk would exist lifelong and does not change with the environment. Yet, in our MR study, there is no reverse causality association to be found between psoriasis and TG.

This study had several strengths. Firstly, two cohorts were served as primary data and secondary data in this study, including UKBB and GLGC. Furthermore, SVMR and MVMR methods were performed to analyze all SNPs of blood lipid in three stages to improve the credibility of the results. The MR analysis showed high consistent results, which added to high reliability relationship between blood lipid and psoriasis. Secondly, multiple MR analytical methods were severed in this study, and the consistent trends of sensitivity analysis indicated high reliability. Thirdly, the effects of diabetes and BMI confounders were excluded, and the results obtained were more reliable. Finally, MR-egger intercept analysis and F statistics were performed for all SNPs in blood lipid to exclude the effect of horizontal pleiotropy and weak instrumental variable bias. Overall, the analysis of multiple data and multiple MR methods improved the confidence of results.

However, there are some limitations in this study. Firstly, the definition of “psoriasis” was broad, included psoriasis subtypes such as psoriasis vulgaris, pustular psoriasis and psoriatic arthritis, among others. Secondly, the association of horizontal pleiotropy could not completely be ruled out, which could be mediated via other causal pathways. Thirdly, the sourcing of two cohorts from different databases may have resulted in data overlap, but larger cohorts may reduce this effect. Finally, the cohorts in this study were European population, caution was warranted before applying the findings to non-European populations.

## Conclusion

MR findings suggest that an increase in the serum level of LDL-C and TG are potential causal risk factors for psoriasis. Mendelian randomization (MR) findings provide genetic evidence for causal link between psoriasis and blood lipid. It may be meaningful to monitor and control blood lipid level for a management of psoriasis patients in clinic.

## Data availability statement

The original contributions presented in the study are included in the article/**Supplementary Material**. Further inquiries can be directed to the corresponding authors.

## Author contributions

WL contributed to the conception and design of the study. YT contributed to the analysis and interpretation of data for the work, and revised the manuscript for the second time. Z-YJ contributed to the data analysis. Z-Y-OZ contributed to write the first draft of the manuscript. All authors contributed to the interpretation of the results and critical revision of the manuscript. All authors contributed to the article and approved the submitted version.
