# Supplementary material for: The relationship between blood lipid and risk of psoriasis: univariable and multivariable Mendelian randomization analysis
Source: Front Immunol. 2023 Jun 22;14:1174998. doi: 10.3389/fimmu.2023.1174998 (PMC10323678; doi:10.3389/fimmu.2023.1174998)
Supplement: Supplementary file 2 [file Table_1.docx]

**Supplementary Table 1: Confounder GWAS summary statistics.**

| Trait | Consortium | Sample Size | PMID | Summary data from: |
| --- | --- | --- | --- | --- |
| BMI | GIANT | 681275 | 30124842 | ieu-b-40 |
| T2D | NA | 655,666 | 30054458 | ebi-a-GCST006867 |

GWAS, genome wide association study; BMI, Body Mass Index; T2D, Type 2 Diabetes; HDL,High-density lipoprotein cholesterol; LDL,Low-density lipoprotein cholesterol; GIANT, Genetic Investigation of ANthropometric Traits GSCAN, GWAS & Sequencing Consortium of Alcohol and Nicotine use
