# Supplementary material for: The relationship between blood lipid and risk of psoriasis: univariable and multivariable Mendelian randomization analysis
Source: Front Immunol. 2023 Jun 22;14:1174998. doi: 10.3389/fimmu.2023.1174998 (PMC10323678; doi:10.3389/fimmu.2023.1174998)
Supplement: Supplementary file 3 [file Table_2.docx]

**Supplementary Table 2: Calculated F-statistics of genetic instruments for lipid traits**

|  | N-SNPs | rsq [%] | sample size | Calculated F-statistics |
| --- | --- | --- | --- | --- |
| *Genetic instruments used in primary database analysis* | | | | |
| LDL-C |  |  |  |  |
| Stage 1 | 193 | 2.83 | 440546 | 66 |
| Stage 2 | 161 | 2.47 | 440546 | 69 |
| Stage 3 | 152 | 2.29 | 440546 | 68 |
| HDL-C |  |  |  |  |
| Stage 1 | 467 | 6.55 | 403943 | 61 |
| Stage 2 | 369 | 5.47 | 403943 | 63 |
| Stage 3 | 337 | 5.00 | 403943 | 63 |
| TG |  |  |  |  |
| Stage 1 | 386 | 5.03 | 441016 | 60 |
| Stage 2 | 295 | 4.09 | 441016 | 64 |
| Stage 3 | 266 | 3.62 | 441016 | 62 |
| *Genetic instruments used in secondary database analysis* | | | | |
| LDL-C |  |  |  |  |
| Stage 1 | 97 | 2.35 | 173082 | 43 |
| Stage 2 | 90 | 2.23 | 173082 | 44 |
| Stage 3 | 80 | 1.87 | 173082 | 41 |
| HDL-C |  |  |  |  |
| Stage 1 | 120 | 2.57 | 187167 | 41 |
| Stage 2 | 108 | 2.39 | 187167 | 42 |
| Stage 3 | 89 | 2.05 | 187167 | 44 |
| TG |  |  |  |  |
| Stage 1 | 69 | 1.66 | 177861 | 43 |
| Stage 2 | 61 | 1.52 | 177861 | 45 |
| Stage 3 | 49 | 1.22 | 177861 | 45 |
