# Supplementary material for: The relationship between blood lipid and risk of psoriasis: univariable and multivariable Mendelian randomization analysis
Source: Front Immunol. 2023 Jun 22;14:1174998. doi: 10.3389/fimmu.2023.1174998 (PMC10323678; doi:10.3389/fimmu.2023.1174998)
Supplement: Supplementary file 4 [file Table_3.docx]

**Supplementary Table 3: 2-sample MR results for associations between lipid traits and psoriasis in primary *database* analysis.**

| Exposure | MR Methods | OR | 95%LCI | 95%UCI | pval | Qhet (pval) | Egger_intercept (pval) |
| --- | --- | --- | --- | --- | --- | --- | --- |
| LDL-C |  |  |  |  |  |  |  |
| Stage 1 | IVW | 1.110 | 0.987 | 1.248 | 0.082 | 6.48E-12 | / |
|  | MR Egger | 1.203 | 1.014 | 1.427 | 0.035 | / | 0.206 |
|  | Weighted median | 1.156 | 1.004 | 1.331 | 0.043 | / | / |
|  | Weighted mode | 1.210 | 1.068 | 1.371 | 0.003 | / | / |
| Stage 2 | IVW | 1.152 | 1.054 | 1.259 | 0.002 | 0.999 | / |
|  | MR Egger | 1.250 | 1.100 | 1.421 | 0.001 | / | 0.083 |
|  | Weighted median | 1.156 | 1.005 | 1.330 | 0.042 | / | / |
|  | Weighted mode | 1.216 | 1.075 | 1.376 | 0.002 | / | / |
| Stage 3 | IVW | 1.145 | 1.039 | 1.261 | 0.006 | 1.000 | / |
|  | MR Egger | 1.268 | 1.101 | 1.461 | 0.001 | / | 0.054 |
|  | Weighted median | 1.342 | 1.137 | 1.584 | 4.98E-04 | / | / |
|  | Weighted mode | 1.270 | 1.091 | 1.478 | 0.002 | / | / |
| HDL-C |  |  |  |  |  |  |  |
| Stage 1 | IVW | 0.917 | 0.825 | 1.020 | 0.112 | 2.65E-41 | / |
|  | MR Egger | 0.985 | 0.836 | 1.161 | 0.857 | / | 0.265 |
|  | Weighted median | 0.945 | 0.823 | 1.086 | 0.427 | / | / |
|  | Weighted mode | 0.905 | 0.793 | 1.033 | 0.141 | / | / |
| Stage 2 | IVW | 0.954 | 0.885 | 1.029 | 0.226 | 1.000 | / |
|  | MR Egger | 0.960 | 0.856 | 1.077 | 0.490 | / | 0.886 |
|  | Weighted median | 0.946 | 0.823 | 1.088 | 0.438 | / | / |
|  | Weighted mode | 0.912 | 0.797 | 1.044 | 0.181 | / | / |
| Stage 3 | IVW | 0.969 | 0.896 | 1.048 | 0.426 | 1.000 | / |
|  | MR Egger | 0.962 | 0.856 | 1.081 | 0.513 | / | 0.869 |
|  | Weighted median | 0.950 | 0.822 | 1.097 | 0.482 | / | / |
|  | Weighted mode | 0.921 | 0.800 | 1.060 | 0.251 | / | / |
| TG |  |  |  |  |  |  |  |
| Stage 1 | IVW | 1.222 | 1.103 | 1.353 | 1.17E-04 | 3.92E-18 | / |
|  | MR Egger | 1.147 | 0.985 | 1.335 | 0.078 | / | 0.272 |
|  | Weighted median | 1.343 | 1.169 | 1.542 | 3.00E-05 | / | / |
|  | Weighted mode | 1.259 | 1.106 | 1.432 | 0.001 | / | / |
| Stage 2 | IVW | 1.146 | 1.057 | 1.243 | 0.001 | 1.000 | / |
|  | MR Egger | 1.192 | 1.059 | 1.342 | 0.004 | / | 0.372 |
|  | Weighted median | 1.342 | 1.172 | 1.536 | 2.09E-05 | / | / |
|  | Weighted mode | 1.333 | 1.162 | 1.530 | 5.33E-05 | / | / |
| Stage 3 | IVW | 1.140 | 1.047 | 1.241 | 0.002 | 1.000 | / |
|  | MR Egger | 1.205 | 1.068 | 1.360 | 0.003 | / | 0.210 |
|  | Weighted median | 1.343 | 1.160 | 1.554 | 7.71E-05 | / | / |
|  | Weighted mode | 1.336 | 1.155 | 1.545 | 1.22E-04 | / | / |
