# Supplementary material for: The relationship between blood lipid and risk of psoriasis: univariable and multivariable Mendelian randomization analysis
Source: Front Immunol. 2023 Jun 22;14:1174998. doi: 10.3389/fimmu.2023.1174998 (PMC10323678; doi:10.3389/fimmu.2023.1174998)
Supplement: Supplementary file 5 [file Table_4.docx]

**Supplementary Table 4: 2-sample MR results for associations between lipid traits and psoriasis in secondary *database* analysis.**

| Exposure | MR Methods | OR | 95%LCI | 95%UCI | pval | Qhet (pval) | Egger_intercept (pval) |
| --- | --- | --- | --- | --- | --- | --- | --- |
| LDL-C |  |  |  |  |  |  |  |
| Stage 1 | IVW | 1.087 | 0.990 | 1.194 | 0.081 | 2.64E-06 | / |
|  | MR Egger | 1.100 | 0.955 | 1.267 | 0.190 | / | 0.826 |
|  | Weighted median | 1.080 | 0.965 | 1.209 | 0.178 | / | / |
|  | Weighted mode | 1.135 | 1.029 | 1.252 | 0.013 | / | / |
| Stage 2 | IVW | 1.099 | 1.024 | 1.180 | 0.009 | 0.580 | / |
|  | MR Egger | 1.122 | 1.009 | 1.247 | 0.036 | / | 0.619 |
|  | Weighted median | 1.088 | 0.970 | 1.219 | 0.149 | / | / |
|  | Weighted mode | 1.142 | 1.037 | 1.258 | 0.009 | / | / |
| Stage 3 | IVW | 1.117 | 1.029 | 1.212 | 0.008 | 0.433 | / |
|  | MR Egger | 1.162 | 1.023 | 1.319 | 0.023 | / | 0.426 |
|  | Weighted median | 1.242 | 1.085 | 1.423 | 0.002 | / | / |
|  | Weighted mode | 1.203 | 1.064 | 1.360 | 0.004 | / | / |
| HDL-C |  |  |  |  |  |  |  |
| Stage 1 | IVW | 0.926 | 0.837 | 1.025 | 0.139 | 4.02E-05 | / |
|  | MR Egger | 0.910 | 0.752 | 1.101 | 0.335 | / | 0.830 |
|  | Weighted median | 0.948 | 0.828 | 1.086 | 0.443 | / | / |
|  | Weighted mode | 0.954 | 0.821 | 1.108 | 0.538 | / | / |
| Stage 2 | IVW | 0.926 | 0.854 | 1.005 | 0.065 | 0.809 | / |
|  | MR Egger | 0.889 | 0.762 | 1.037 | 0.136 | / | 0.538 |
|  | Weighted median | 0.948 | 0.826 | 1.089 | 0.451 | / | / |
|  | Weighted mode | 0.964 | 0.827 | 1.124 | 0.642 | / | / |
| Stage 3 | IVW | 0.933 | 0.856 | 1.016 | 0.111 | 0.651 | / |
|  | MR Egger | 0.898 | 0.766 | 1.053 | 0.189 | / | 0.581 |
|  | Weighted median | 0.950 | 0.823 | 1.096 | 0.479 | / | / |
|  | Weighted mode | 0.971 | 0.833 | 1.131 | 0.704 | / | / |
| TG |  |  |  |  |  |  |  |
| Stage 1 | IVW | 1.224 | 1.063 | 1.409 | 0.005 | 4.02E-07 | / |
|  | MR Egger | 1.161 | 0.927 | 1.454 | 0.199 | / | 0.555 |
|  | Weighted median | 1.204 | 1.033 | 1.405 | 0.018 | / | / |
|  | Weighted mode | 1.212 | 1.043 | 1.407 | 0.014 | / | / |
| Stage 2 | IVW | 1.173 | 1.062 | 1.296 | 0.002 | 0.731 | / |
|  | MR Egger | 1.203 | 1.027 | 1.409 | 0.025 | / | 0.685 |
|  | Weighted median | 1.202 | 1.027 | 1.406 | 0.022 | / | / |
|  | Weighted mode | 1.211 | 1.029 | 1.425 | 0.025 | / | / |
| Stage 3 | IVW | 1.137 | 1.018 | 1.271 | 0.023 | 0.544 | / |
|  | MR Egger | 1.193 | 1.001 | 1.421 | 0.055 | / | 0.496 |
|  | Weighted median | 1.185 | 1.009 | 1.391 | 0.039 | / | / |
|  | Weighted mode | 1.151 | 0.973 | 1.360 | 0.107 | / | / |
