# Supplementary material for: The relationship between blood lipid and risk of psoriasis: univariable and multivariable Mendelian randomization analysis
Source: Front Immunol. 2023 Jun 22;14:1174998. doi: 10.3389/fimmu.2023.1174998 (PMC10323678; doi:10.3389/fimmu.2023.1174998)
Supplement: Supplementary file 6 [file Table_5.docx]

| Outcome | N-SNP | MR Methods | Beta | 95%LCI | 95%UCI | pval | Qhet (pval) | Egger_intercept (pval) |
| --- | --- | --- | --- | --- | --- | --- | --- | --- |
| LDL-C | 27 | IVW | -0.009 | -0.016 | -0.002 | 0.009 | 0.008 | / |
|  |  | MR Egger | -0.014 | -0.027 | 0.000 | 0.061 | / | 0.461 |
|  |  | Weighted median | -0.013 | -0.021 | -0.005 | 0.001 | / | / |
|  |  | Weighted mode | -0.017 | -0.028 | -0.005 | 0.008 | / | / |
| HDL-C | 21 | IVW | -0.011 | -0.021 | -0.002 | 0.016 | 4.26E-06 | / |
|  |  | MR Egger | -0.021 | -0.039 | -0.004 | 0.028 | / | 0.209 |
|  |  | Weighted median | -0.018 | -0.027 | -0.009 | 4.62E-05 | / | / |
|  |  | Weighted mode | -0.018 | -0.028 | -0.008 | 0.002 | / | / |
| TG | 27 | IVW | -0.007 | -0.015 | 0.000 | 0.056 | 1.28E-04 | / |
|  |  | MR Egger | -0.013 | -0.028 | 0.002 | 0.101 | / | 0.404 |
|  |  | Weighted median | -0.008 | -0.016 | -0.001 | 0.035 | / | / |
|  |  | Weighted mode | -0.012 | -0.024 | 0.000 | 0.063 | / | / |

**Supplementary Table 5: reverse MR results for associations between psoriasis and lipid traits.**
